# Integration of Metabolomics With Pharmacodynamics to Elucidate the Anti-myocardial Ischemia Effects of Combination of Notoginseng Total Saponins and Safflower Total Flavonoids

**DOI:** 10.3389/fphar.2018.00667

**Published:** 2018-06-25

**Authors:** Yuqing Meng, Zhiyong Du, Yan Li, Lichao Wang, Peng Gao, Xiaoyan Gao, Chun Li, Mingbo Zhao, Yong Jiang, Pengfei Tu, Xiaoyu Guo

**Affiliations:** ^1^State Key Laboratory of Natural and Biomimetic Drugs, School of Pharmaceutical Sciences, Peking University, Beijing, China; ^2^School of Chinese Material Medica, Beijing University of Chinese Medicine, Beijing, China; ^3^Modern Research Center for Traditional Chinese Medicine, Beijing University of Chinese Medicine, Beijing, China

**Keywords:** metabolomics, myocardial ischemia, cardioprotective effects, UPLC-Q-TOF/MS, notoginseng total saponins, safflower total flavonoids

## Abstract

Notoginseng (Sanqi), the roots and rhizomes of *Panax notoginseng* and safflower, the flowers of *Carthamus tinctorius*, are widely used traditional Chinese medicines (TCMs) for the treatment of cardiovascular diseases. Positive evidences have fueled growing acceptance for cardioprotective effects of the combination of the notoginseng total saponins and safflower total flavonoids (CNS) against myocardial ischemia (MI). However, the underlying cardioprotective mechanisms of CNS are still obscured. Metabolomics is a comprehensive tool for investigating biological mechanisms of disease, monitoring therapeutic outcomes, and advancing drug discovery and development. Herein, we investigated the cardioprotective effects of CNS on the isoproterenol (ISO)-induced MI rats by using plasma and urine metabolomics based on ultra-performance liquid chromatography coupled with quadrupole-time of flight mass spectrometry (UPLC-Q-TOF/MS) and multiple pharmacodynamics approaches. The results showed that pretreatment with CNS could attenuate the cardiac injury resulting from ISO, as evidenced by decreasing the myocardial infarct size, converting the echocardiographic, histopathological, and plasma biochemical abnormalities, and reversing the perturbations of plasma and urine metabolic profiles, particularly for the 55.0 mg/kg dosage group. In addition, 44 metabolites were identified as the potential MI biomarkers, mainly including a range of free fatty acids (FFAs), sphingolipids, and glycerophospholipids. CNS pretreatment group may robustly ameliorate these potential MI-related biomarkers. The accumulation of LysoPCs and FFAs, caused by PLA_2_, may activate NF-κB pathway and increase proinflammatory cytokines. However, our results showed that CNS at 55.0 mg/kg dosage could maximally attenuate the NF-κB signaling pathway, depress the expressions of TNF-α, IL-6, IL-1β, and PLA_2_. The results suggested that the anti-inflammatory property of CNS may contribute to its cardioprotection against MI. Our results demonstrate that the integrating of metabolomics with pharmacodynamics provides a reasonable approach for understanding the therapeutic effects of TCMs and CNS provide a potential candidate for prevention and treatment of MI.

## Introduction

Cardiovascular diseases (CVDs) are the leading cause of morbidity and mortality worldwide and MI is the most predominant presentation of CVDs (Dominguez-Rodriguez et al., 2014). MI is characterized by an acute condition of ischemic necrosis of heart muscle that occurs as a result of the interruption in the supply of myocardial oxygen and nutrients (Zhou et al., 2008). Overstimulation of isoproterenol (ISO), a synthetic β-adrenergic receptor agonist was reported to induce MI through severe cardiac stress, including the highly cytotoxic free radicals producing, intracellular Ca^2+^ overload, and apoptosis (Garson et al., 2015). The experimental model of ISO-induced MI offered a reliable technique to investigate the efficacy of various potentially cardioprotective agents, which has been frequently documented in previous studies (Fathiazad et al., 2012). MI is a devastating disease which is an urgent need for medical breakthroughs. TCM plays a key role on people’s health care from ancient to modern times in China. Today, TCM has been accepted worldwide as a powerful alternative for acute or chronic disorders. For example, CDDP, a commonly used TCM patent drug to treat CVDs in China has been approved by Food and Drug Administration (FDA) of United States for stages II and III Investigational New Drug (IND) examinations (Sun et al., 2013; Luo et al., 2015).

Notoginseng (Sanqi), the roots and rhizomes of *Panax notoginseng* (Burk.) F. H. Chen (PN) and safflower, the flowers of *Carthamus tinctorius* L. are two popularly used TCMs in China and other Asian countries. The combination of these two herbal drugs has been usually applied in a number of TCM prescriptions for the prevention and treatment of CVDs in clinic. It has been confirmed that the saponins and flavonoids are the major bioactive components of PN and safflower, respectively (Chen et al., 2014, 2016). Notoginseng total saponins could inhibit the release of myocardial enzymes and reduce myocardial infarction in MI rats. Additionally, safflower total flavonoids could limit infarct size and improve cardiac function in MI rats (Han et al., 2013). Our previous studies (Han et al., 2010, 2011) presented that CNS, which mainly contained notoginsenoside R_1_, ginsenoside Rg_1_, ginsenoside Re, ginsenoside Rb_1_, ginsenoside Rd, hydroxysafflor yellow A, and kaempferol-3-*O*-rutinoside, exhibited significant cardioprotective effects *in vivo*. However, the cardioprotective mechanism of CNS has not been fully elucidated.

Metabolomics which aims at systematically analyzing the changes of endogenous metabolites in a biological system before and after inner or external disturbances has been applied in many fields. Traditional analysis strategy is not sufficient to elucidate the mechanisms of TCM due to its multi-components and complex therapeutic effects. However, metabolomics appears ideal for TCM holistic philosophy and has been successfully applied to illustrate the mechanisms of many TCMs with cardioprotective activities and to seek the specific disease-related metabolites (Qi et al., 2013; Chen et al., 2015; Yang et al., 2015; Zou et al., 2015). In addition, metabolomics provides a valuable assistance with pharmacodynamics research. The combination of metabolomics and pharmacodynamics provide an excellent chance to understand the therapeutic effects and evaluate the efficacy of TCM.

In this study, the electrocardiography, histopathological examination, myocardial infarct size, and biochemical analysis were performed to evaluate the efficacy of CNS on the ISO-induced MI rats. Meanwhile, plasma and urine metabolomics approaches based on UPLC-Q-TOF/MS were designed to assess the cardioprotective effects of different dosages of CNS. Finally, immunohistochemistry assay of NF-κB signaling pathway and PLA_2_ were performed to elucidate the potential anti-inflammation properties of CNS.

## Materials and Methods

### Chemicals and Reagents

Acetonitrile (LC grade) and methanol (LC grade) were purchased from Merck (Darmstadt, Germany). Formic acid (LC grade) was purchased from Fisher Scientific (Spain). Ultrapure water (18.2 MΩ) was prepared using a Milli-Q water purification system (Millipore, MA, United States). Other chemicals were of analytical grade and their purity was above 99.5%. Arachidonic acid, docosahexaenoic acid, palmitoleic acid, corticosterone, sphingosine, and sphinganine were obtained from Cayman Chemical (Michigan, United States). Isoproterenol, TTC, leucine enkephalin, L-phenyl-*d_5_*-alanine, 4-pyridoxic acid, citric acid, sebacic acid, uric acid, palmitic acid, L-palmitoylcarnitine, stearidonic acid, L-tryptophan, oleic acid, pantothenate, and sphingosine-1-phosphate were purchased from Sigma-Aldrich (St. Louis, MO, United States). Palmitic acid, LysoPC (19:0), and LysoPC (16:0) were purchased from Avanti Polar Lipids, Inc. (Alabaster, United States). Ascorbic acid, 3′, 5′-Cyclic AMP, inosine, suberic acid, nalidixic acid, aldosterone, chenodeoxycholic acid, linoleic acid, and phytosphingosine were purchased from J&K SCIENTIFIC LTD. (Beijing, China). Poly formaldehyde was purchased from Beijing Solarbio Science & Technology Co., Ltd. (Beijing, China). Compound Danshen Dripping Pill (CDDP) was purchased from Tianjin Tasly Pharmaceutical Co., Ltd. (Tianjin, China). Ultrapure water (18.2 MΩ) was prepared using a Milli-Q water purification system (Millipore, United States). All the antibodies used in immunohistochemistry were purchased from Abcam, United States.

Notoginseng total saponins (NS) was purchased from Yunnan Plant Pharmaceutical Co., Ltd. (Kunming, China). The quality standard of NS complied with Chinese Pharmacopoeia (2010 edition) and the content of notoginsenoside R_1_, ginsenoside Rg_1_, ginsenoside Re, ginsenoside Rb_1_, and ginsenoside Rd was 6.2%, 26.6%, 4.1%, 32.5%, and 6.6%, respectively. Safflower was collected from Xinjiang Uygur Autonomous Region (China), and authenticated by Prof. Pengfei Tu. The voucher specimen of safflower (No. 20110301) was deposited in Modern Research Center for Traditional Chinese Medicine, Peking University (Beijing, China). The materials of safflower were refluxed with deionized water at 80°C for three times (120 L for 1 h, 100 L for 0.5 h, and 100 L for 0.5 h). The extract was filtered and concentrated in vacuo. The concentrated solution was subjected to a D101 macroporous resin column eluted with deionized water and 50% aqueous ethanol. The 50% aqueous ethanol eluate was dried by spray drying to obtain SF. The content of the effective components (total safflor yellow and total flavonoids) in SF was more than 40% by ultraviolet-visible spectrophotometry. Meanwhile, the content of hydroxysafflor yellow A and kaempferol-3-*O*-rutinoside in SF were more than 8.0% and 0.20% by high performance liquid chromatography, respectively. The ratio of NS and SF was 6:5 in CNS.

### Animals

Thirty-six Sprague-Dawley rats (240 ± 10 g male) were provided by the Department of Laboratory Animal Science, Peking University Health Science Center (Beijing, China) and were fed a certified standard diet and water. The temperature and humidity were set at 23 ± 2°C and 40–60%, respectively. A 12 h light/dark cycle was used.

### Animal Study

After acclimatization, the animals were randomly classified into six groups with eight rats each: normal group, model group, the low dose of CNS (L-CNS) group, the middle dose of CNS (M-CNS) group, the high dose of CNS (H-CNS) group and positive group. Rats in L-CNS, M-CNS, and H-CNS groups were administered CNS via oral gavage at the doses of 27.5, 55.0, and 82.5 mg/kg, respectively. Rats in normal and model groups received an oral administration of saline water for seven consecutive days. The rats in MI, L-CNS, M-CNS, H-CNS, and positive group were subcutaneously injected with isoproterenol (100.0 mg/kg, once daily) on days 8 and 9, and the rats in normal group were subcutaneously injected with the equal volume of normal saline for 2 consecutive days. The rats in positive group received CDDP at 107.0 mg/kg via oral gavage, an optimal effective dose that had been verified in an MI rat model (Xin et al., 2013; Zou et al., 2015). A 24-h urine sample on the 10th day of each animal was collected in an individual metabolism cage and centrifuged at 3,500 rpm and 4°C for 15 min to remove the particle contaminants. The urinary supernatants were collected and stored at -80°C. After urine collection, all the rats were anesthetized with sodium pentobarbital (50.0 mg/kg), and the blood samples were collected into the heparinized tubes. and the plasma were obtained by centrifuging for 10 min at 3,500 rpm and 4°C, the obtained supernatants were stored at -80°C. The hearts were harvested immediately washed with cold physiological saline and stored at -80°C for further analysis.

### Electrocardiography, Histology and Immunohistochemistry Assay

Twenty-four hours after the last dose of ISO, subcutaneous peripheral limb electrodes were inserted under the skin of the anesthetized rats. Twelve-lead ECG recordings were continued for at least 5 min using BL-420F Data Acquisition & Analysis System (Techman soft, Chengdu, China). Heart tissue samples were fixed in 4% poly formaldehyde solution at room temperature for 24 h after a brief rinse with PBS, and the heart tissue specimens were subsequently embedded in paraffin. The paraffin sections, approximately 3 μm, were stained with routine H&E staining for histological examination. Immunohistochemistry was performed using antibodies against COX-2, IKK-β, IL-6, NF-κB, iNOS, TNF-α, TRAF-6, and PLA_2_. Photographs were obtained by a light microscope IX73 (Olympus, Germany) at 400 magnifications.

### TTC Staining to Determine Infarction Area

After all rats were sacrificed, their hearts were immediately removed and placed at -20°C for 10 min, then each was cut into five pieces. They were incubated for 20 min in 1% TTC solution at 37°C. The infarcted tissues remained white, whereas normal tissues were stained red. The infarct zone was analyzed by Image-Pro Plus image analysis software (NIH Image, version 1.61). To assess the cardioprotective effects of CNS, IS/LV was calculated.

### Biochemical Analysis

For cardiac enzymology assays, the plasma concentrations of CK and LDH were measured via an ultraviolet spectrophotometer using commercial kits (Jiancheng Bioengineering Institute, Nanjing, China). cTnT was determined by an enzyme-linked immunosorbent assay using a commercially available standard enzymatic kit (Cusabio Biotech Co., Ltd., Wuhan, China). For oxidative stress parameters, the levels of SOD and MDA in plasma were spectrophotometrically measured using diagnostic kits (Jiancheng Bioengineering Institute, Nanjing, China). For inflammatory cytokines assay, the levels of TNF-α, IL-6, and IL-1β were determined by an enzyme-linked immunosorbent assay using a commercially available standard enzymatic kit (Cusabio Biotech Co., Ltd., Wuhan, China). Significance of the data was analyzed by a two-tailed Student’s *t*-test with a critical *p*-value of 0.05.

### Sample Preparation

#### Rat Plasma Sample Preparation

The plasma samples were thawed at 4°C. A volume of 150 μL of ice-cold methanol-acetonitrile (1:1, v/v) containing 0.1 mg/mL L-phenyl-*d_5_*-alanine and 0.1 mg/mL LysoPC (19:0) as the internal standards was added to a 50 μL aliquot of plasma to precipitate the proteins. After vortex-blending for 1 min and incubation on ice for 5 min, the mixture was centrifuged at 14,000 rpm for 10 min at 4°C. A volume of 150 μL supernatant was transferred into a clean dry tube and evaporated to dryness at 30°C. The dried residue was reconstituted in 80 μL ice-cold solvents [a mixture of water-methanol-acetonitrile (10:3:3, v/v/v)] and centrifuged at 14,000 rpm for 5 min at 4°C before UPLC-Q-TOF/MS analysis.

#### Rat Urine Sample Preparation

The urine samples were unfrozen at 4°C, and a volume 400 μL of ice-cold methanol-ultrapure water (3:7, v/v) containing 0.1 mg/mL L-phenyl-*d_5_*-alanine as the internal standard was added to precipitate protein, after vortex-mixing for 1 min and incubation on ice for 5 min. It was centrifuged at 14,000 rpm for 10 min. A volume 400 μL supernatant was transferred into a clean tube before metabolomics analysis. The “pooled” QC sample was prepared following the procedure of preparation described above. All samples were random coded and subjected to UPLC-Q-TOF/MS analysis. The QC sample was injected every 6 real samples throughout the entire experiment.

### UPLC-Q-TOF/MS Analysis

Metabolomics analysis was performed on a Waters ACQUITY UPLC system coupled with a dual electrospray ionization probe and a Micromass QTOF micro Synapt High Definition Mass Spectrometer from Waters (Waters Corporation, Milford, MA, United States). For the analysis of plasma samples, the chromatographic separation was through an ACQUITY UPLC BEH C18 column (2.1 mm × 50 mm, 1.7 μm, Waters Corporation, Milford, MA, United States). The column was maintained at 45°C, and the flow rate was 0.4 mL/min as 2 μL aliquot of each sample was injected. The optimal mobile phase consisted of a linear gradient system of (A) 0.1% formic acid in water and (B) 0.1% formic acid in acetonitrile: 0-1.0 min, 3-20% B; 1.0-6.0 min, 20-60% B; 6.0-9.5 min, 60% B; 9.5-11.5 min, 60-90% B; 11.5-13.5 min, 90-100% B; 13.5-15.5 min, 100% B; 15.5-16.5 min, 100-3% B; 16.5-18.5 min, 3% B. The parameters of the MS detection were as follows: both positive and negative ion modes were employed in the operation of plasma samples, and an electrospray ionization source (ESI) was applied; the source temperature was set at 110°C; the desolvation gas temperature and desolvation gas flow were 500°C and 700 L/h, respectively; the capillary voltage was 3.2 kV for positive ion mode (ESI+) and 2.0 kV for negative ion model (ESI-); sampling cone voltage was 35 V; the extraction cone voltage was 1.0 V; the cone gas rate was set at 50 L/h.

For analysis of the urine samples, the metabolite separation was performed on an ACQUITY UPLC T3 column (2.1 mm × 50 mm, 1.7 μm, Waters Corp., Milford, MA, United States). The column oven was maintained at 40°C, and the flowing rate was set at 0.4 mL/min as 2 μL aliquot of each sample was injected. The mobile phase consisted of (A) water with 0.1% formic acid and (B) acetonitrile with 0.1% formic acid. The following linear gradient conditions were set as follows: 0-2.5 min, 0-2% B; 2.5-5.5 min, 2-26% B; 5.5-8 min, 26–50% B; 8-9.5 min, 50–70% B; 9.5-10.5 min, 70-100% B; 11.5-12.5 min, 100-0% B; and 12.5-15 min, 0% B. The conditions of the MS analysis were as follows: the source temperature was set at 110°C. The desolvation gas temperature and desolvation gas flow were 500°C and 800 L/h, respectively, the capillary voltage was 3.2 kV for ESI+ and 2.2 kV for ESI-, sampling cone voltage was 35 V, the extraction cone voltage was 1.0 V, the cone gas rate was set at 50 L/h.

All analyses were acquired using a LockSpray interface to ensure the accuracy and reproducibility. Leucine-enkephalin was used as the reference compound with *m/z* 556.2771 for ESI+ and 554.2615 for ESI-. Data were collected in centroid mode from 100 to 1,000 Da. The MS/MS analyses of the ions were performed at different collision energy parameters that ranged from 5 and 50 eV for plasma samples and from 10 and 50 eV for the urine samples. The representative BPI chromatograms of the rat plasma and urine obtained in ESI negative and positive mode are illustrated in **Supplementary Figure S1**.

### Sample Repeatability and System Stability

The relative standard derivations (RSDs) of retention times and peak areas for five selected ions which selected according to different chemical polarities and *m/z* values in six batches of plasma and urine samples were employed to evaluate the samples repeatability. The RSDs for retention times of the selected ions were less than 0.61% and 0.78% in positive and negative modes, respectively. The RSDs for peak areas of the selected ions were less than 9.42% and 10.43% in positive and in negative modes, respectively. The QC sample was injected into LC-MS every 6 real samples throughout the entire experiment to evaluate the LC-MS system stability. The PCA score plot and validation data of the QC samples showed good system stability. The RSDs for the retention times of the five peaks in plasma samples were less than 0.84% in positive mode and less than 0.96% in negative mode, and the RSDs for the peak areas were less than 9.25% and 8.76% in positive mode and negative mode, respectively. The stable retention times for five ions in urine QC samples were also observed with the RSDs less than 0.45% and 0.62% for negative mode and positive mode, respectively. In addition, the RSDs for peak areas of the selected ions were less than 10.31% in positive mode and less than 11.47% in negative mode in urine QC samples.

### Metabolomic Data Processing and Biomarker Identification

The raw data were analyzed using MarkerLynx Applications Manager Version 4.1 (Waters, Manchester, United Kindom) for deconvolution, alignment, and data reduction. The MVA for the data matrix was performed using SIMCA-P software (v14.0, Umetric, Umeå, Sweden). The quality of the MVA models was controlled by evaluating the *R*^2^ and *Q*^2^ values. An unsupervised PCA was firstly employed to give the comprehensive view of the clustering trends for the group separation and to assess the quality, homogeneity, and outlier identification of the dataset. Subsequently, a supervised orthogonal partial least squares discriminate analysis (OPLS-DA) was performed to discriminate the separation in the established PCA model and search the potential biomarkers. The Pareto scaling was employed in both PCA and OPLS-DA. The *p*-values of CV-ANOVA were used to test the validity of the OPLS-DA model against overfitting. The *S*-plot (an absolute *p* (corr) > 0.4) and the VIP plot (VIP > 1.5) based on the OPLS-DA model was combined to identify the significantly altered ions as potential biomarkers for MI diagnosis. The selected variables were subsequently confirmed by the Student’s *t*-test (*p* < 0.05) and the univariate ROC curve by using the area under the ROC curve (AUC) (cutoff AUC area value ≥ 0.8).

For the identification of potential biomarkers, several online databases, such as HMDB^1^, METLIN^2^, LIPIDMAPS^3^, and KEGG^4^ were used by comparing the exact molecular mass and fragments information obtained from UPLC-Q-TOF/MS. The matched metabolite was further identified by comparing with the retention time and MS/MS fragmentation patterns of the authentic standards. Based on the identified and validated potential biomarkers, an unsupervised Heatmap was constructed to explore the correlations and visualization of the hierarchical relationship among the groups by using the online MetaboAnalyst^5^. A metabolic pathway analysis facilitating further biological interpretation of the identified potential biomarkers was performed using MetPA to reveal the most relevant pathways. For the parameters of pathway analysis algorithms, hypergeometric test was used for over representation analysis, and relative-between centrality was used for pathway topology analysis.

## Results

### CNS Prevented the Electrocardiogram Changes in MI Rats

**FIGURE 1:**
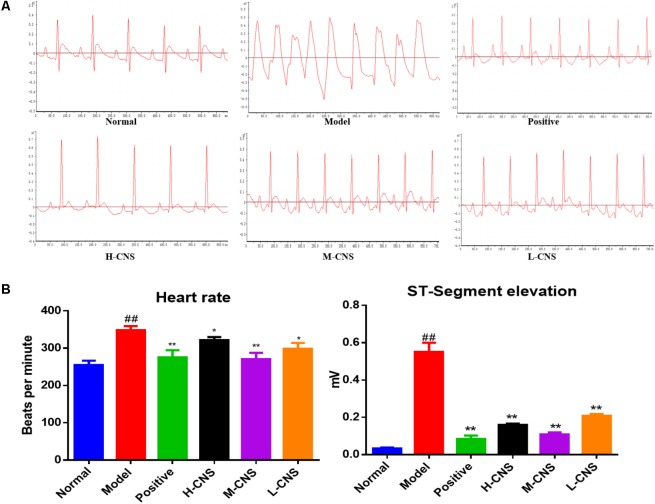
**(A)** Representative ECG of rats in different groups. **(B)** Effects of CNS on isoproterenol-induced electrocardiographic changes. Values expressed as the mean ± SD. Significance was assessed with a two-tailed Student’s *t*-test: ^##^*p* < 0.01 vs. the normal group; ^∗^*p* < 0.05, ^∗∗^*p* < 0.01 vs. the model group.

The ST segment drifts on electrocardiogram were firstly examined in ISO-induced MI rats. Representative traces of twelve-lead ECG waveforms were shown in **Figure 1A**. At the end of the experiment, rats treated with isoproterenol demonstrated an increase in ST segments and QRS complex and a decrease in QT intervals in comparison with the normal group. Oral administration of CNS at 27.5, 55.0, and 82.5 mg/kg effectively prevents these above changes of electrocardiogram parameters. As a positive control, CDDP at 107.0 mg/kg showed similar beneficial effects on ECG parameters (**Figure 1B**).

### CNS Attenuated the Histopathological Damages of Myocardium Under MI Condition

**FIGURE 2:**
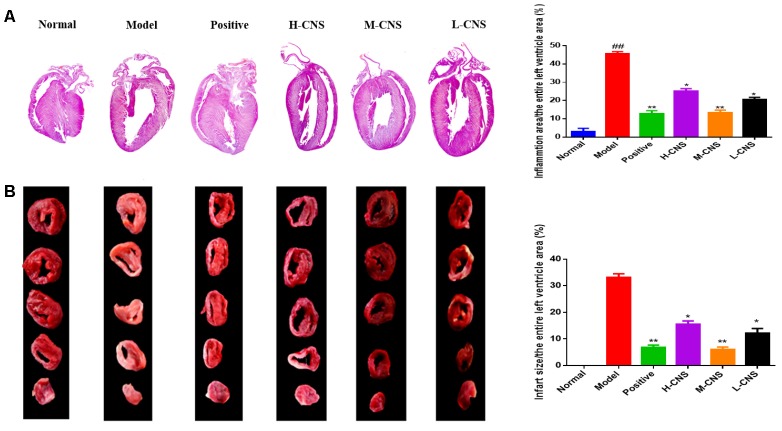
**(A)** Representative photomicrographs of cardiac tissue sections stained with H&E, (*n* = 3/group). Values expressed as the mean ± SD. Significance was assessed with a two-tailed Student’s *t*-test: ^##^*p* < 0.01 vs. the normal group; ^∗^*p* < 0.05, ^∗∗^*p* < 0.01 vs. the model group. **(B)** Infarct size of rats in each group assessed by TTC assay. The infarcted tissues remained white, whereas normal tissues were stained red (*n* = 3/group). Values expressed as the mean ± SD. Significance was assessed with a two-tailed Student’s *t*-test: ^∗^*p* < 0.05, ^∗∗^*p* < 0.01 vs. the model group.

**Figure 2A** showed photographs for cardiac sections stained with H&E. Moreover, the pathological changes in different groups were also demonstrated in **Figure 2A**. Normal rats evidenced that the myocardial fibers were arranged regularly with clear striations and no apparent degeneration, necrosis, and inflammatory cells, whereas histological sections in the model group showed loss of striations, confluent necrosis, edema, and inflammatory cell infiltration. However, pre-treatment with CNS and positive drug significantly inhibited the isoproterenol-induced histopathological changes in the heart tissues. Importantly, treatment with the M-CNS showed normal myocardial architectures with obvious transverse striations, decreased fibrosis grade and less inflammatory cells.

### CNS Reduced Myocardial Infarct Size

The most widely used approach to differentiate viable tissue from infarcted tissue macroscopically is TTC staining. As illustrated in **Figure 2B**, myocardial infarct size of each group was assessed by TTC staining method. Furthermore, the percentage of IS/LV was counted to estimate the cardioprotective effects of CNS. Compared with the normal group, there was a significantly increase of myocardial infarct size in the model group, and the percentage of IS/LV was over 30% in the model group. Pretreatment with CNS and positive drug can pronouncedly attenuate the myocardial infarct size induced by ISO, particularly for the positive group and the M-CNS group.

### Therapeutic Efficacy of CNS

**FIGURE 3:**
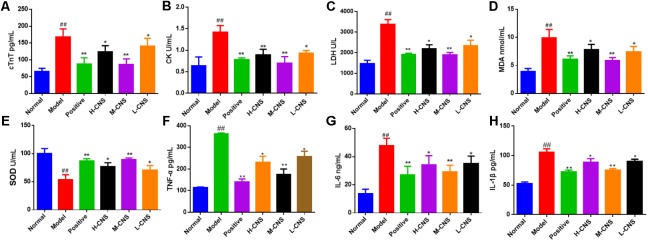
Effects of CNS on plasma biochemical indicators in MI rats. **(A)** cTnT; **(B)** CK; **(C)** LDH; **(D)** MDA; **(E)** SOD; **(F)** TNF-α; **(G)** IL-6; **(H)** IL-1β. Values expressed as the mean ± SD. Significance was assessed with a two-tailed Student’s *t-*test: ^##^*p* < 0.01 vs. the normal group; ^∗^*p* < 0.05, ^∗∗^*p* < 0.01 vs. the model group (*n* = 6).

It is well-known that cTnT, CK, and LDH are the reliable myocardial biomarkers for MI. As shown in **Figures 3A–C**, the plasma concentrations of these myocardial enzymes were significantly (*p* < 0.05) increased in the model group as compared with the normal group. Furthermore, the plasma levels of MDA and SOD were assessed and expressed as oxidative pathogenesis indicators in the present study. The MI rats showed a significant decrease in the level of SOD (*p* < 0.05) and a remarkable increase in the level of MDA (*p* < 0.05) compared with the normal group (**Figures 3D,E**). Moreover, the levels of TNF-α, IL-6, and IL-1β were also expressed as the indicator of inflammation progress responses to ISO, and significant increase of TNF-α, IL-6, and IL-1β was shown in the MI rats (*p* < 0.05) compared with the normal rats. Pretreatment with CNS and positive drug can significantly reverse TNF-α, IL-6, and IL-1β alterations in plasma (**Figures 3F–H**). It is worth pointing out that the pretreatment with M-CNS can significantly reverse all these biochemical parameters near to the normal levels, indicating that CNS is capable of ameliorating the acute cellular necrosis, easing oxidative stress and anti-inflammation.

### Metabolomics Analysis of the Plasma and Urine Samples

**FIGURE 4:**
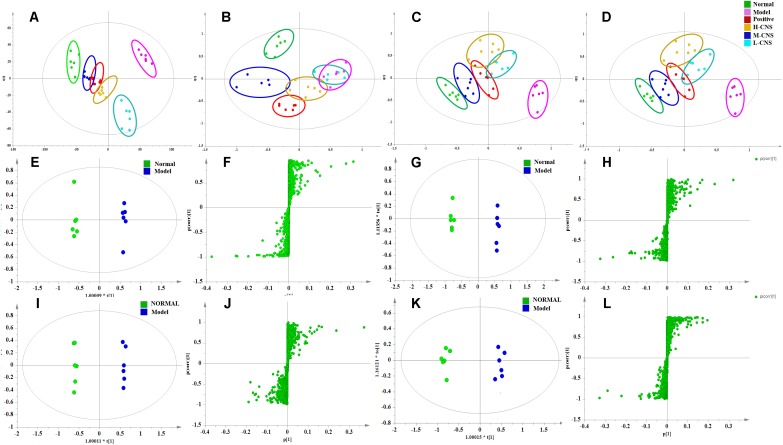
Pattern analysis of data from the metabolic profiles of plasma and urine by UPLC-Q-TOF/MS. **(A)** PCA scores plot of the plasma samples in positive mode. **(B)** PCA scores plot of the plasma samples in negative mode. **(C)** PCA scores plot of the urine samples in positive mode. **(D)** PCA scores plot of the urine samples in negative mode. **(E)** OPLS-DA score plot of plasma samples from normal and MI model rats in positive mode. **(F)** *S*-plot from OPLS-DA of plasma samples from normal and MI model rats in positive mode. **(G)** OPLS-DA score plot of plasma samples from normal and MI model rats in negative mode. **(H)** *S*-plot from OPLS-DA of plasma samples from normal and MI model rats in negative mode. **(I)** OPLS-DA score plot of urine samples from normal and MI model rats in positive mode. **(J)** *S*-plot from OPLS-DA of urine samples from normal and MI model rats in positive mode. **(K)** OPLS-DA score plot of urine samples from normal and MI model rats in negative mode. **(L)** *S*-plot from OPLS-DA of urine samples from normal and MI model rats in negative mode.

The PCA is the most frequently adopted approach to distinguish among the classes in metabolomics fields. As shown in **Figures 4A–D**. the score plots indicated that the metabolic states of the plasma and urine of rats in the model group deviated from the normal group, suggesting that significant biochemical changes were induced by ISO. The metabolic profiles of rats in the M-CNS pre-treated groups considerably differed from the model group, and located much closer to the normal group than the positive group, indicating the biochemical alterations induced by MI were significantly inhibited after the pretreatment of CNS. However, the L-CNS and H-CNS groups were close to each other and away from the normal group, and the L-CNS group had a much shorter distance to the model group than the H-CNS group. Similar group classification was observed both in the positive pattern of the plasma and urine samples and the negative pattern of the plasma samples, except in the negative pattern of the urine samples, because the L-CNS and the model groups showed projections in nearly the same area. Metabolomic profiling results suggested the M-CNS group had a preferable cardioprotective effect than the H-CNS and L-CNS groups, which were consistent with the pathological and biochemical findings. PCA score plot of the QC samples and tested samples in ESI negative and positive mode were shown in **Supplementary Figure S2**.

### CNS Improved the Potential Biomarkers Related to MI

Efficient diagnostic metabolites are needed for effectively assessment of global metabolic profiles between the MI and the normal rats and accurately evaluation of the therapeutic effects of drugs. OPLS-DA method was employed to sharpen the established separation between the model and the normal groups in PCA pattern and identify the significantly altered variables responsible for differentiation. As expected, a remarkable separation between the MI and the normal rats was observed in the OPLS-DA score plot (**Figures 4E–L**). The CV-ANOVA *p*-value (4.431^E-18^) suggests that the OPLS-DA model is highly significant and implied non-overfitting. According to the previously described protocol, 12 metabolites in urine and 32 metabolites in plasma that contributed to the separation of the MI and the normal rats were selected and identified as the significantly altered metabolic biomarkers for MI (**Supplementary Table S1**). The dominant metabolites present in plasma mainly included a range of polyunsaturated fatty acids, saturated fatty acids, sphingolipids, LysoPCs, phosphatidylcholine (PCs), lysophosphatidylethanolamines (LysoPEs), and phatidylethanolamines (PEs). The altered metabolites in urine included several fatty acids, 3′,5′-cyclic AMP, citric acid, inosine, and uric acid. These potential biomarkers were primarily distributed in energy metabolism, lipid metabolism, oxidative injury, and inflammation response (**Supplementary Figure S3**). To narrow down the scope of the potential biomarkers pool, the hierarchical cluster analysis based on their Spearman correlation coefficients was employed to reveal the potential relationships among them. The results were presented in the hierarchical cluster plot with different colors (**Supplementary Figure S4**). Linoleic acid, arachidonic acid, 9, 12, 13-TriHOME, 9, 10, 13-TriHOME, chenodeoxycholic acid, and docosahexaenoic acid were collected in one cluster, all of those metabolites were involved in arachidonic acid metabolism; sphinganine, sphingosine, and phytosphingosine, which associated with sphingolipid metabolism, were collected in one cluster; LysoPC (16:0), LysoPC (18:2), LysoPC (18:1), LysoPC (22:6), and LysoPC (20:3) were clustered closely; and LysoPE (20:1), PE (22:2/15:0), and PE (18:1/22:5) were collected in one cluster, which belonged to glycerophospholipid metabolism. The result indicated that the similar type metabolites were distributed in the same cluster and had similar changing trends.

**FIGURE 5:**
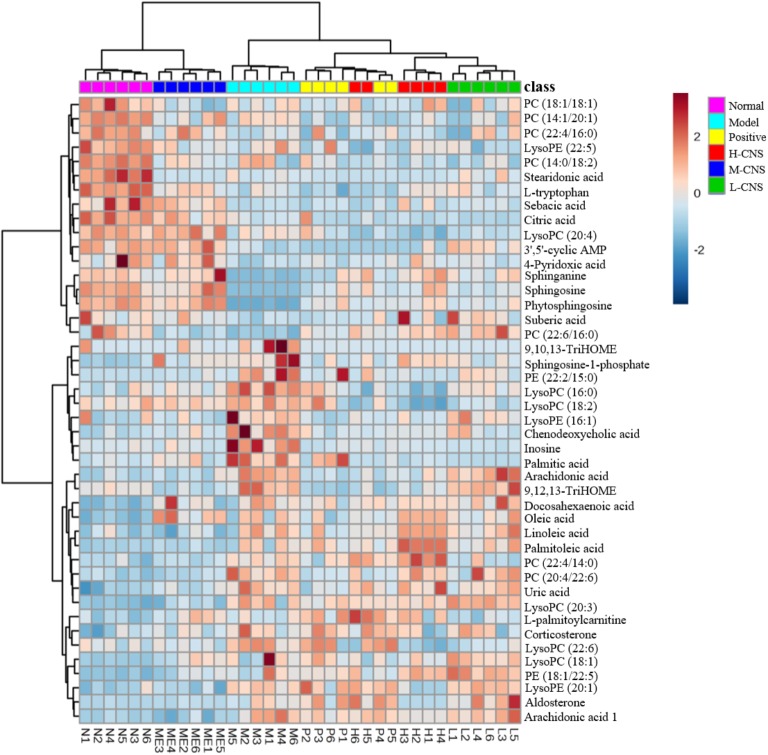
Heatmap of potential biomarker intensities in different groups.

To further investigate the recovery condition of these 44 bio-candidates by CNS, the relative peak areas among three CNS-dosed, positive drug-dosed, and model groups were tested via univariate ROC curve analyses and Student’s *t*-tests. **Supplementary Table S1** presented the changed trends of these potential biomarkers in details. On the whole, after the different doses of CNS and the positive drug pretreatments, the levels of these metabolites were regulated to some degree. The intensities of these potential biomarkers in the M-CNS and normal groups exhibited similar patterns, which were distinct from the model and the other treatment groups, as shown in the Heatmap visualization (**Figure 5**). Of particularly, the potential biomarkers related with inflammation, such as arachidonic acid, chenodeoxycholic acid, inosine, sphinganine, sphingosine, LysoPC (16:0), LysoPC (18:2), LysoPC (22:6), PC (22:6/16:0), LysoPE (16:1), and palmitic acid, returned to normal degree after the M-CNS treatment.

### CNS Inhibited the MI-Induced Myocardial Inflammation

**FIGURE 6:**
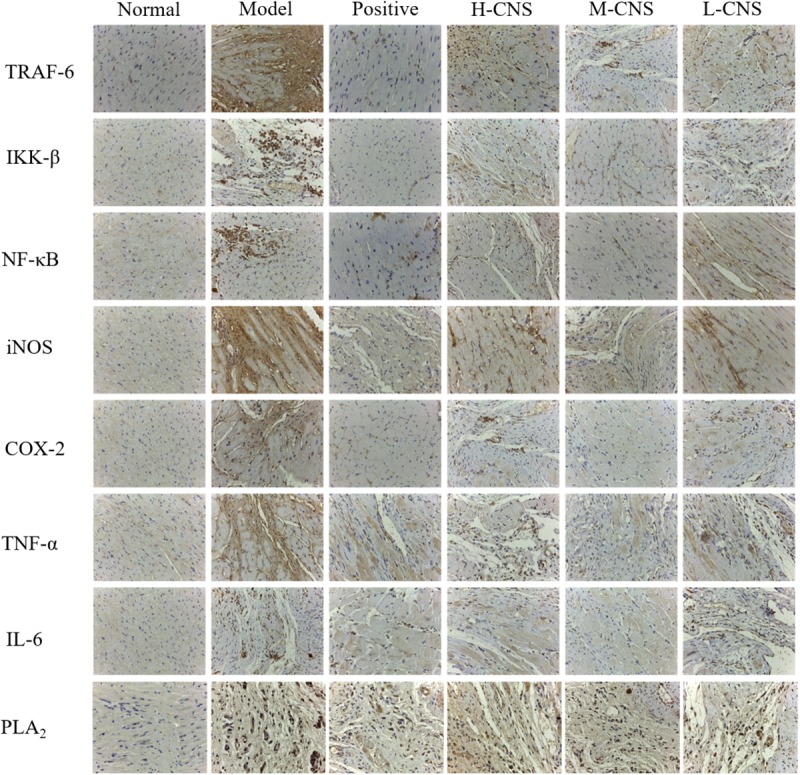
Representative immunohistochemistry photomicrographs.

Isoproterenol treated myocardium showed marked inflammation in H&E observation, and the above metabolomics results also indicated that a considerable number of altered metabolites in MI rats were also closely linked with inflammation. Thus, we adopted the immunohistochemistry approach to study the expression levels of various inflammatory cytokines and the PLA_2_ in the heart tissues. NF-κB signaling pathway played a crucial role in immune regulation, inflammation, stress response and apoptosis. In this research, we observed apparent overexpression of COX-2, IKK-β, IL-6, NF-κB, iNOS, TNF-α, and TRAF6 (**Figure 6**). Meanwhile, **Figure 6** illustrated the expression of PLA_2_ in different groups. The level of PLA_2_ in the model group remarkably up-regulated compared with the normal group. Pretreatment with CNS significantly attenuated the over-expression of PLA_2_, particularly for the M-CNS group. Moreover, the plasma levels of TNF-α, IL-6, and IL-1β were also expressed as the indicator of inflammation progress responses to ISO, and the MI rats also showed a significant increase in the TNF-α, IL-6, and IL-1β level (*p* < 0.05) compared with the normal rats. As expected, all these pro-inflammatory cytokines were significantly ameliorated in the CNS and positive drug pretreatment rats, particularly in the M-CNS group.

## Discussion

With the advent and development of metabolomic platform, metabolomics has been shown to be a highly specific and useful tool for the diagnosis of disease and evaluation of drug efficacy, which also ultimately resulted a considerable process in the definition of MI and drug discovery and development. Although used for thousands of years, the underlying mechanisms of TCM therapeutic efficacies remain poorly understood as a result of its multi-component and multi-target regulation.

**FIGURE 7:**
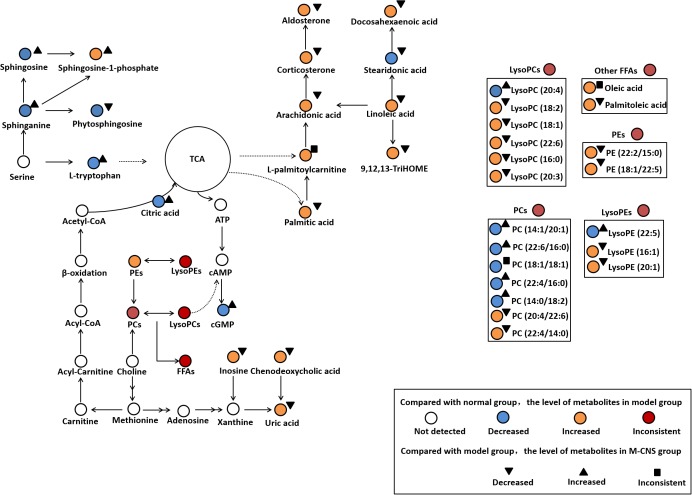
Summary of the cardioprotective effects of CNS on the network of vital potential biomarkers in MI rats.

In the present study, the ECG, plasma biochemistry, histopathological examinations, and TTC staining confirmed that the different doses of CNS pretreatment exhibited considerable therapeutic efficacies on ISO-induced MI injuries. Of particular note was that the pretreatment with M-CNS substantially attenuated the elevation of ST-segments in ECG, lowered the plasma levels of cTnT, CK, and LDH, and inhibited the histopathological damages in MI rats. Previous study reported that the extracts of PN and safflower possessed anti-inflammatory ability (Chang et al., 2007; Jun et al., 2011). Moreover, in present study of immunohistochemistry assay of NF-κB signaling pathway and the PLA_2_, pretreatment of M-CNS also showed a prominent anti-inflammatory efficacy. However, a relatively slighter reverse was observed in the pretreatments of H-CNS and L-CNS. In line with the results of pharmacodynamics study, metabolomic profile analysis found that M-CNS pretreatment can maximally retain their plasma and urinary baseline levels close to those of the normal group, demonstrating that CNS could effectively prevent ISO-induced myocardial disorders. According to the heatmap analysis, the beneficial effects of M-CNS were further validated by reversing potential biomarkers close to normal levels. Collectively, we built the metabolomic features network depicting the protection of CNS against MI (**Figure 7**). Network reconstruction has led to the integration of these altered metabolites associated with the caused perturbation of multiple pathways, especially for the potential biomarkers in the pathways associated with inflammation responses, oxidative damage, apoptosis, and energy metabolism disorders.

L-palmitoylcarnitine, a major long chain acylcarnitine, has been generally considered as a negative effector during MI. The accumulation of L-palmitoylcarnitine during hypoxia and ischemia may lead to the increased ROS generation and the intracellular Ca^2+^ overload (Suzuki et al., 1993; Netticadan et al., 1999), and cause mitochondrial dysfunction and ventricular dysfunctions (Roussel et al., 2015). L-palmitoylcarnitine can also enhance the release of arachidonic acid during ischemia (Boeynaems et al., 1989). Linoleic acid, the precursor of many inflammatory molecules, (Gilbert et al., 2016), can induce vasoconstriction in coronary arteries. Linoleic acid and arachidonic acid, as liberated polyunsaturated fatty acids (PUFAs), are substrates of lipoxygenases and activated by cell injury. 9, 12, 13-TriHOME is a trihydroxyoctadecenoic acid metabolite of linoleic acid, which may be involved in regulating prostaglandin synthesis (Funk and Powell, 1983). Increased plasma level of aldosterone has been associated with large-scale clinical trials in patients with a variety of CVD states, including acute myocardial infarction (Cohn and Colucci, 2006), atherosclerosis (De Rita et al., 2012), and heart failure (Maron et al., 2016). Aldosterone exerts numerous detrimental effects on the cardiovascular system including inflammation, oxidative stress, apoptosis, and fibrosis (Beygui et al., 2010; Weir et al., 2011). CNS pretreatment could repair the inflammation-related disorders of linoleic acid, arachidonic acid, 9, 12, 13-TriHOME and aldosterone in MI rats, particularly in the M-CNS rats.

It has been shown that FFAs can increase the expression of proinflammatory cytokines and activate canonical proinflammatory NF-κB pathway *in vivo* and *in vitro* studies. Acute increases in plasma FFA levels activated NF-κB pathway, and increased the expressions of several proinflammatory cytokines including TNF-α, IL-6, and IL-1β (Zeng et al., 2015). In the present study, FFAs such as arachidonic acid, linoleic acid, and palmitic acid were significantly increased in the model group compared to the normal group. Nevertheless, the CNS pretreatment could antagonize the increased plasma levels of a variety of LysoPCs and FFAs after ISO stimulation. Based on the discovery of metabolomics, the immunohistochemistry assay of NF-κB signaling pathway and the levels of TNF-α, IL-6, and IL-1β in plasma were performed to strengthen the metabolomic findings and verify the anti-inflammation properties of CNS through NF-κB pathway. Moreover, previous studies have shown that the accumulation of LysoPCs and FFAs is a result of increased degradation of membrane phosphatidylcholines by PLA_2_ enzymes (Nanda et al., 2007). Thus, the immunohistochemistry assay of PLA_2_ in heart tissues were also performed, and the results showed that the model group was characterized with higher expressions of PLA_2_ compared to the normal group. However, pretreatment with CNS could significantly inhibit the expressions of PLA_2_ after ISO induction. Our results identified that CNS protected against ISO-induced myocardial inflammation via the NF-κB pathway by decreasing the expressions of PLA_2_, levels of LysoPCs and FFAs, particularly in the M-CNS.

Previous study Chiva-Blanch et al. (2016) demonstrated a higher percentage of serum oleic acid in acute myocardial infarction patients, and the elevated plasma oleic acid may cause endothelial dysfunction and atherosclerosis through sequential activation of protein kinase C (PKC) and NF-κB-dependent signaling pathways, which showed a highly correlation with inflammation (Park et al., 2003). Moreover, palmitic acid, a 16-carbon saturated fatty acid, could induce apoptosis in endothelial cells via p38 and JNK mitogen-activated protein kinase pathways (Jiang et al., 2010), and it can activate NF-κB-dependent signaling pathways to stimulate inflammatory responses (Handl et al., 2018). Palmitic acid may also serve as a source of ROS through mitochondrial oxidation (Stentz and Kitabchi, 2006). Increasing evidence suggested a positive association between the plasma palmitoleic acid and the risk of coronary heart disease (Djousse et al., 2012). Additionally, palmitoleic acid, a monounsaturated fatty acid could induce mitochondrial dysfunction in the heart (Oyanagi et al., 2015), cardiac arrhythmias and atherosclerosis (Merino et al., 2015). In our study, high levels of oleic acid, palmitic acid, and palmitoleic acid were observed in the MI rats and were down-regulated to normal by CNS pretreatment, particularly for the M-CNS group. These findings revealed that CNS might protect the cardiomyocytes after ISO stimulation by inhibiting apoptosis-related metabolites.

L-tryptophan is an important energy metabolism precursor of the tricarboxylic acid (TCA) cycle (Knapp et al., 1986). L-tryptophan as an antioxidant could regress the hypoxic myocardial injury (Narin et al., 2010). Citric acid was reported to have cardioprotective effect on MI through anti-inflammatory, antiplatelet aggregation and anti-apoptotic mechanisms (Tang et al., 2013). High level of uric acid may exert a negative effect on the pathogenesis of CVDs by stimulating inflammation and oxidative stress (Wang R. et al., 2016; Liu et al., 2017). Increased circulating uric acid level is recognized as a marker associated with acute ST-segment elevation myocardial infarction in patients (Omidvar et al., 2012; Lazaros et al., 2013). Numerous studies have demonstrated that the enhanced inosine acts as a biomarker for acute cardiac ischemia, myocardial infarction in patients and animal models (Fazekas et al., 1999; Farthing et al., 2007, 2015). Inosine is produced in cardiomyocytes and released in large qualities under hypoxia, ischemia and other forms of cellular stress. It augments myocardial blood flow and increases contractility of ischemia heart muscle (Czarnecki et al., 1992). These significantly altered metabolites were strongly associated with energy metabolism disorder and oxidative damage occurring after ISO stimulation. The M-CNS pretreatment could effectively ameliorate the increased levels of uric acid and inosine in MI rats, and reverse the decreased levels of L-tryptophan and citric acid to meet the energy need in the organism.

Sphingolipids are major constituents of cell membranes and are present at high concentrations in blood. In growing recent researches, sphingolipids attracted much attention and have been implicated in both pathophysiology of CVDs and cardioprotective actions (Alewijnse and Peters, 2008; Jiang and Liu, 2013). Sphinganine is the substrate for the synthesis of sphingosine and phytosphingosine (Crossman and Hirschberg, 1984; Knapp et al., 2013). Sphingosine at a high level could decrease the cardiac contractility and induce the apoptosis during ischemia/reperfusion (Alewijnse and Peters, 2008). Phytosphingosine could induce apoptosis by activation of caspase 3 and release of cytochrome c (Liu et al., 2014). Sphingosine-1-phosphate (S1P) is a sphingolipid metabolite formed by phosphorylation of sphingosine in a reaction catalyzed by the enzyme sphingosine kinase. Interestingly, an increased S1P content contributes to cardiac inflammation, remodeling and dysfunction following MI. S1P is able to assist TNF-α signaling through TNF-α associated receptor leading to subsequent NF-κB activation (Zhang et al., 2016). Our results demonstrated that TNF-α, NF-κB, and TRAF6 expression were increased in the heart tissues of MI rats. Moreover, a clinical study indicated that the significantly increased plasma S1P was a novel potential marker for early detection of myocardial ischemic injury (Egom et al., 2013). In the present study, the plasma levels of sphinganine, sphingosine, and sphingosine-1-phosphate were increased in model group compared to the normal group. CNS pretreatment significantly decreased the levels of sphinganine, sphingosine and S1P, especially for the M-CNS and positive groups.

Glycerophospholipids are key components of the lipid bilayers and important signaling molecules, involving in a wide range of pathophysiological processes, such as proliferation, inflammation, and apoptosis. Moreover, diverse structurally phospholipids in plasma have been recognized to be biomarkers for different types of CVDs (Meikle et al., 2011; Stegemann et al., 2014; Takeda et al., 2015; Fan et al., 2016; Sutter et al., 2016). Previous metabolomics researches on the animals of ISO-induced MI also demonstrated a significant permutation in plasma and serum phospholipids (Wang Z. et al., 2016; Zhou et al., 2017). Our results indicated that the plasma LysoPC (16:0), LysoPC (18:2), LysoPC (18:1), LysoPC (22:6), and LysoPC (20:3) levels exhibited a strong positive association with ISO induced MI for most species with the exception of LysoPC (20:4). LysoPC (16:0) could enhance the expression of MCP-1 and activate the transcription factor NF-κB pathway, promote lipid peroxidation and MDA accumulation (Zurgil et al., 2007). LysoPCs containing unsaturated fatty acids (sn1 linked) showed a strongest association to MI. It has been recently discovered that the increased LysoPCs may induce inflammation, apoptosis and cardiac contractile dysfunction in CVDs (Chen et al., 1997; Xu et al., 2016). In general, LysoPCs and free acids (e.g., arachidonic acid) can be released through hydrolysis of the ester bond of PCs (Slone and Fleming, 2014). In the present study, we also found that the alterations in PCs, which possessed anti-oxidation, anti-inflammation and anti-fibrosis activities. PC (22:4/16:0) and PC (22:4/14:0) were increased, and PC (22:6/16:0), PC (18:1/18:1), and PC (14:1/20:1) were decreased in plasma of model rats compared with the normal rats. The increased LysoPC (18:1) and LysoPC (22:6) might contribute to the decreased PC (22:6/16:0) and PC (18:1/18:1) under cardiac stress. LysoPEs, considered being important arrhythmogenic lipids, have been reported to be increased in the infarcted myocardium of rats with MI induced by coronary artery ligation (Mastroroberto et al., 1996; Menger et al., 2012). In our findings, the plasma LysoPE (16:1), LysoPE (22:5), and LysoPE (20:1) were significantly enhanced in the MI rats compared with the normal group. However, the M-CNS pretreatment could maintain the normal levels of those LysoPCs, PCs and LysoPEs in plasma, indicating CNS may maintain the stability of glycerophospholipids levels against the disorders of glycerophospholipids metabolism during MI.

Most of the CNS targeted-metabolites included FFAs, citric acid, uric acid, S1P, and LysoPCs were mainly associated with the anti-inflammation responses. The results of immunohistochemistry assay of NF-κB signaling pathway and PLA_2_ were consistent with multiple pharmacodynamics and metabolomics. All these highly expressed pro-inflammatory cytokines were ameliorated in the CNS pretreatment rats, particularly in the M-CNS group.

In summary, our research highlights the role of metabolomics to elucidate plasma and urine metabolic characters of ISO-induced MI and monitor the therapeutic effects of different doses of CNS at the global metabolomics and specific biomarkers levels. Of note, the potential biomarkers and major pathways were mainly associated with several highly expressed pro-inflammatory cytokines, indicating that the inflammation occurring after ISO stimulation. Our results indicated that CNS pretreatment could provide satisfactory therapeutic effects on MI through regulating multiple perturbed potential biomarkers, attenuating the NF-κB signaling pathway, depressing the expressions of proinflammatory cytokines, and PLA_2_ to their normal state.

## Ethics Statement

All procedures followed the relevant national legislation and were approved by the Institutional Animal Care and Use Committee of Peking University Health Science Center (No. LA2015061).

## Author Contributions

XGu, YJ, and PT designed the research. YM, YL, and CL performed the animal experiments. PG and LW performed the pharmacodynamic studies. YM, ZD, MZ, and XGa performed the mass spectrometry and data analysis. YM and ZD performed the statistical tests and drafted the manuscript. XGu finalized the writing. All authors read and approved the final manuscript.

## Conflict of Interest Statement

The authors declare that the research was conducted in the absence of any commercial or financial relationships that could be construed as a potential conflict of interest. The reviewer JC declared a shared affiliation, with no collaboration, with the authors XGa and CL to the handling Editor.

## Footnotes

1 http://www.hmdb.ca/

2 http://metlin.scripps.edu/

3 http://www.lipidmaps.org/

4 www.genome.jp/kegg/

5 http://www.metaboanalyst.ca/

## Abbreviations

CDDP: Compound Danshen Dropping Pill
CK: creatine kinase
CNS: the combination of notoginseng total saponins and safflower total flavonoids
COX-2: cyclo-oxygenase 2
cTnT: cardiac troponin T
CV-ANOVA: cross-validated analysis of variance
CVDs: cardiovascular diseases
ECG: electrocardiography
FFAs: free fatty acids
H&E: hematoxylin and eosin
IKK-β: inhibitor of nuclear factor kappa-B kinase
IL-1β: interleukin-1β
IL-6: interleukin-6
iNOS: inducible nitric oxide synthase
IS/LV: infarct size to the entire left ventricle area
LDH: lactate dehydrogenase
LysoPCs: lysophosphatidylcholines
MCP-1: monocyte chemoattractant protein 1
MDA: malondialdehyde
MetPA: Metabolomics Pathway Analysis
MI: myocardial ischemia
MVA: multivariate statistical analysis
NF-κB: nuclear factor kappa-light-chain-enhancer of activated B cells
OPLS-DA: orthogonal partial least squares discriminate analysis
PBS: phosphate buffered saline
PCA: principal component analysis
PLA_2_: phospholipase A_2_
QC: quality control
ROC: receiver operating characteristic
SOD: superoxide dismutase
TCM: traditional Chinese medicine
TNF-α: tumor necrosis factor-α
TRAF6: TNF receptor associated factor 6
TTC: 2, 3, 5-triphenyltetrazoliumchoride
UPLC-Q-TOF/MS: ultra-performance liquid chromatography coupled with quadrupole-time of flight mass spectrometry
VIP: variable influence in the projection
