# Supplementary material for: Integration of Metabolomics With Pharmacodynamics to Elucidate the Anti-myocardial Ischemia Effects of Combination of Notoginseng Total Saponins and Safflower Total Flavonoids
Source: Front Pharmacol. 2018 Jun 25;9:667. doi: 10.3389/fphar.2018.00667 (PMC6026671; doi:10.3389/fphar.2018.00667)
Supplement: Supplementary file 1 [file Data_Sheet_1.docx]

Supplementary Material

**Integration of metabolomics with** **pharmacodynamics to elucidate the anti-myocardial ischemia effects of combination of notoginseng total saponins and safflower total flavonoids**

***Yuqing Meng ^1#^, Zhiyong Du ^1#^, Yan Li ^1^, Lichao Wang ^1^, Peng Gao ^1^, Xiaoyan Gao ^2^, Chun Li ^3^, Mingbo Zhao ^1^, Yong Jiang ^1^, Pengfei Tu ^1^, Xiaoyu Guo ^1*^***

*^1^ State Key Laboratory of Natural and Biomimetic Drugs, School of Pharmaceutical Sciences, Peking University, Beijing 100191, China*

*^2^ School of Chinese Material Medica, Beijing University of Chinese Medicine, Beijing University of Chinese Medicine, Beijing 100029, China*

*^3^ Modern Research Center for Traditional Chinese Medicine, Beijing University of Chinese Medicine, Beijing 100029, China*

^#^ These authors contributed equally to this work.

*** Correspondence:**

*Xiaoyu Guo*

[*guoxiaoyu@bjmu.edu.cn*](mailto:guoxiaoyu@bjmu.edu.cn)

# 1. Supplementary Figures and Tables

**Supplementary Figure 1.** The representative base peak intensity (BPI) chromatograms of the rat plasma and urine samples in ESI negative and positive mode by UPLC-Q-TOF/MS. (A) BPI chromatogram of the plasma sample in positive mode; (B) BPI chromatogram of the plasma sample in negative mode; (C) BPI chromatogram of the urine sample in positive mode; (D) BPI chromatograms of the urine sample in negative mode.

**Supplementary Figure 2.** PCA score plot of the QC and tested samples in ESI negative and positive mode by UPLC-Q-TOF/MS.

**Supplementary Figure 3.** Summary of the metabolic pathway enrichment analysis in MetaboAnalyst.

**Supplementary Figure 4.** Hierarchical cluster analysis based on Spearman correlation coefficients of the potential biomarkers in rat plasma and urine.

**Supplementary Table 1.** Identification of the potential biomarkers in rat plasma and urine by UPLC-Q-TOF/MS.

## 1.1 Supplementary Figures

**Supplementary Figure 1.** The representative base peak intensity (BPI) chromatograms of the rat plasma and urine samples in ESI negative and positive mode by UPLC-Q-TOF/MS. (A) BPI chromatogram of the plasma sample in positive mode; (B) BPI chromatogram of the plasma sample in negative mode; (C) BPI chromatogram of the urine sample in positive mode; (D) BPI chromatograms of the urine sample in negative mode.

**Supplementary Figure 2.** PCA score plot of the QC and tested samples in ESI negative and positive mode by UPLC-Q-TOF/MS. (A) plasma samples; (B) urine samples. The QC samples are marked in in green circles, and the tested samples are marked in blue circles.

**
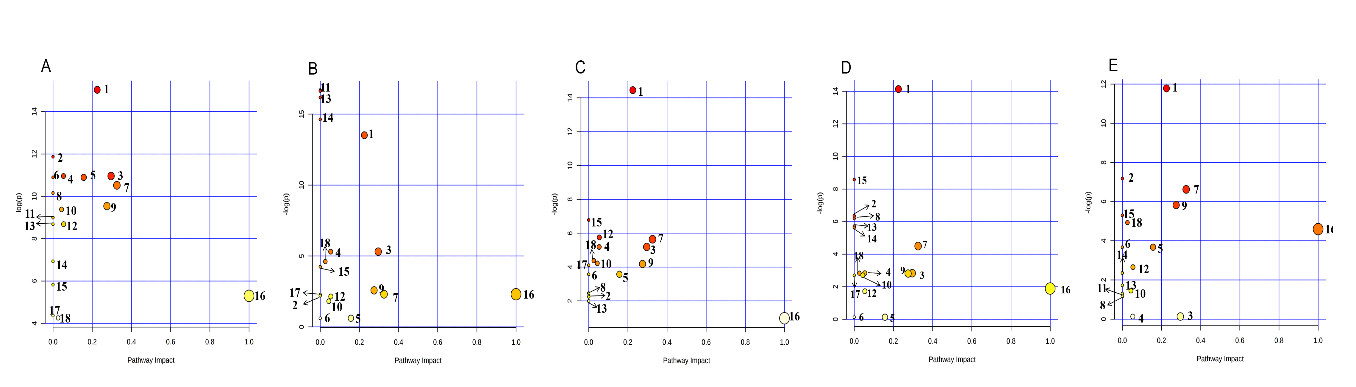
**

**Supplementary Figure 3.** Summary of the metabolic pathway enrichment analysis in MetaboAnalyst. (A) MI target metabolic pathway alterations; (B) Pathways influenced by the L-CNS group; (C) Pathways influenced by the M-CNS group; (D) Pathways influenced by the H-CNS group; (E) Pathways influenced by the positive drug. Interpretation: 1. Sphingolipid metabolism; 2. alpha-Linolenic acid metabolism; 3. Glyoxylate and dicarboxylate metabolism; 4. TCA cycle; 5. Tryptophan metabolism; 6. Aminoacyl-tRNA biosynthesis; 7. Arachidonic acid metabolism; 8. Biosynthesis of unsaturated fatty acids; 9. Glycerophospholipid metabolism; 10. Glycosylphosphatidylinositol(GPI)-anchor biosynthesis; 11. Fatty acid biosynthesis; 12. Steroid hormone biosynthesis; 13. Fatty acid elongation in mitochondria; 14. Fatty acid metabolism; 15. Primary bile acid biosynthesis; 16. Linoleic acid metabolism; 17. Vitamin B6 metabolism; 18. Purine metabolism.


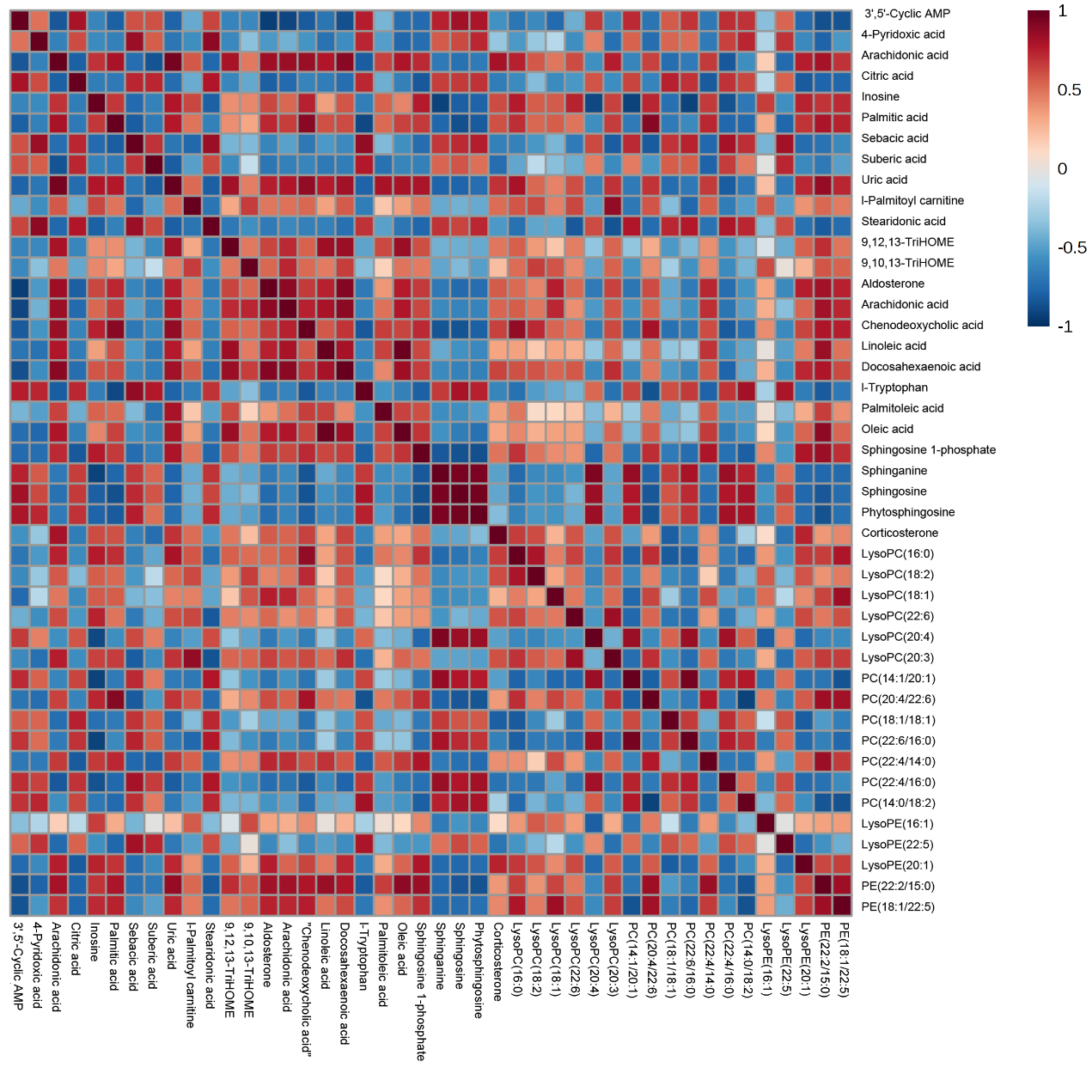


**Supplementary Figure 4.** Hierarchical cluster analysis based on Spearman correlation coefficients of the potential biomarkers in rat plasma and urine.

## 1.2 Supplementary Table

**Supplementary Table 1.** Identification of the potential biomarkers in rat plasma and urine by UPLC-Q-TOF/MS

| **R.T.**  **(min)** | **Exact mass (*m/z*)** | **Formula** | **Detected mode** | **Identification** | **HMDB IDs** | **Normal vs. Model** | **Positive vs.**  **Model** | **H-CNS vs.**  **Model** | **M-CNS vs.**  **Model** | **L-CNS vs. Model** |
| --- | --- | --- | --- | --- | --- | --- | --- | --- | --- | --- |
| 0.80 | 192.0191 | C_6_H_8_O_7_ | - | Citrate *^u,a^* | HMDB00094 | **↓**^Δ^** | ^Δ^- | ^Δ^- | **↑**^Δ^** | **↓**^Δ^** |
| 0.83 | 168.0365 | C_5_H_4_N_4_O_3_ | + | Uric acid *^u,a^* | HMDB00289 | **↑**^Δ^** | **↓**^Δ^** | **↓**^Δ^** | **↓**^Δ^** | /- |
| 0.84 | 168.0206 | C_5_H_4_N_4_O_3_ | - | Uric acid *^u,a^* | HMDB00289 | **↑**^Δ^** | **↓**^Δ^** | **↓**^Δ^** | **↓**^Δ^** | /- |
| 1.09 | 204.0819 | C_11_H_12_N_2_O_2_ | - | L-tryptophan *^p,a^* | HMDB00929 | **↓**^Δ^** | /- | **↓**^Δ^** | **↑**^Δ^** | /- |
| 1.48 | 256.0980 | C_16_H_31_O_2_ | - | Palmitic acid *^p,a^* | HMDB00220 | **↑**^Δ^** | **↓**^Δ^** | /- | **↓**^Δ^** | **↓**^Δ^** |
| 1.79 | 399.1606 | C_23_H_45_NO_4_ | + | L-palmitoylcarnitine *^u,a^* | HMDB00222 | **↑**^Δ^** | **↓**^Δ^** | **↑**^Δ^** | /- | /- |
| 2.11 | 183.0453 | C_8_H_9_NO_4_ | - | 4-Pyridoxic acid *^u,a^* | HMDB00017 | **↓**^Δ^** | **↑**^Δ^** | ^Δ^- | **↑**^Δ^** | ^Δ^- |
| 2.49 | 329.0446 | C_10_H_12_N_5_O_6_P | - | 3',5'-Cyclic AMP *^u,a^* | HMDB00058 | **↓**^Δ^** | **↑**^Δ^** | **↓**^Δ^** | **↑**^Δ^** | **↑**^Δ^** |
| 3.38 | 346.2222 | C_21_H_30_O_4_ | + | Corticosterone *^p,a^* | HMDB01547 | **↑**^Δ^** | /- | ^Δ^- | ^Δ^- | /- |
| 4.26 | 360.1896 | C_21_H_28_O_5_ | - | Aldosterone *^p,a^* | HMDB00037 | **↑**^Δ^* | /- | ^Δ^- | **↓**^Δ^** | /- |
| 4.63 | 392.2850 | C_24_H_40_O_4_ | - | Chenodeoxycholic acid *^p,a^* | HMDB00518 | **↑**^Δ^** | **↓**^Δ^** | **↓**^Δ^** | **↓**^Δ^** | **↓**^Δ^** |
| 4.70 | 317.3016 | C_18_H_39_NO_3_ | + | Phytosphingosine *^p,a^* | HMDB04610 | **↓**^Δ^** | **↑**^Δ^** | **↑**^Δ^** | **↑**^Δ^** | **↑**^Δ^** |
| 4.78 | 299.2905 | C_18_H_37_NO_2_ | + | Sphingosine *^p,a^* | HMDB00252 | **↓**^Δ^** | **↑**^Δ^** | **↑**^Δ^** | **↑**^Δ^** | **↑**^Δ^** |
| 5.32 | 174.0815 | C_8_H_14_O_4_ | - | Suberic acid *^u,a^* | HMDB00893 | **↓**^Δ^** | /- | /- | **↑**^Δ^** | **↑**^Δ^** |
| 5.67 | 301.3061 | C_18_H_39_NO_2_ | + | Sphinganine *^p,a^* | HMDB00269 | **↓**^Δ^** | **↑**^Δ^** | **↑**^Δ^** | **↑**^Δ^** | **↑**^Δ^** |
| 5.77 | 379.2411 | C_18_H_38_NO_5_P | - | Sphingosine-1-phosphate *^p,a^* | HMDB00277 | **↑**^Δ^** | **↓**^Δ^** | /- | /- | /- |
| 6.44 | 567.3404 | C_30_H_50_NO_7_P | + | LysoPC (22:6) *^p^* | HMDB10404 | **↑**^Δ^** | **↓**^Δ^** | /- | **↓**^Δ^** | **↓**^Δ^** |
| 6.46 | 519.3408 | C_26_H_50_NO_7_P | + | LysoPC (18:2) *^p^* | HMDB10386 | **↑**^Δ^** | **↓**^Δ^** | /- | **↓**^Δ^** | **↓**^Δ^** |
| 6.47 | 543.3407 | C_28_H_50_NO_7_P | + | LysoPC (20:4) *^p^* | HMDB10395 | **↓**^Δ^** | **↑**^Δ^** | /- | **↑**^Δ^** | **↓**^Δ^** |
| 6.51 | 527.3096 | C_27_H_45_NO_7_P | + | LysoPE (22:5) *^p^* | HMDB11524 | **↓**^Δ^** | /- | /- | /- | /- |
| 6.55 | 202.1127 | C_10_H_18_O_4_ | - | Sebacic acid *^u,a^* | HMDB00792 | **↓**^Δ^** | /- | /- | **↑**^Δ^** | ^Δ^- |
| 6.89 | 451.2777 | C_21_H_42_NO_7_P | + | LysoPE (16:1) *^p^* | HMDB11504 | **↑**^Δ^* | **↓**^Δ^** | **↓**^Δ^* | **↓**^Δ^** | /- |
| 6.90 | 495.3414 | C_24_H_50_NO_7_P | + | LysoPC (16:0) *^p,a^* | HMDB10382 | **↑**^Δ^** | **↓**^Δ^** | **↓**^Δ^** | **↓**^Δ^** | **↓**^Δ^** |
| 7.25 | 521.3562 | C_26_H_52_NO_7_P | + | LysoPC (18:1) *^p^* | HMDB02815 | **↑**^Δ^** | /- | /- | /- | **↓**^Δ^** |
| 7.46 | 268.1230 | C_10_H_12_N_4_O_5_ | - | Inosine *^u,a^* | HMDB00195 | **↑**^Δ^** | **↓**^Δ^** | **↓**^Δ^** | **↓**^Δ^** | **↓**^Δ^** |
| 7.47 | 330.2326 | C_18_H_34_O_5_ | - | 9,10,13-TriHOME *^u^* | HMDB04710 | **↑**^Δ^* | **↓**^Δ^** | **↓**^Δ^** | ^Δ^- | **↓**^Δ^** |
| 8.89 | 545.3535 | C_28_H_52_NO_7_P | + | LysoPC (20:3) *^p^* | HMDB10393 | **↑**^Δ^** | /- | /- | **↓**^Δ^** | /- |
| 8.91 | 330.2328 | C_18_H_34_O_5_ | - | 9,12,13-TriHOME *^u^* | HMDB04708 | **↑**^Δ^* | **↓**^Δ^** | **↓**^Δ^** | **↓**^Δ^** | /- |
| 8.97 | 507.3407 | C_25_H_50_NO_7_P | + | LysoPE (20:1) *^p^* | HMDB11512 | **↑**^Δ^** | /- | /- | **↓**^Δ^** | /- |
| 9.47 | 276.2169 | C_18_H_28_O_2_ | + | Stearidonic acid *^u,a^* | HMDB06547 | **↓**^Δ^** | **↑**^Δ^** | ^Δ^- | ^Δ^- | /- |
| 10.83 | 254.2168 | C_16_H_29_O_2_ | - | Palmitoleic acid *^p,a^* | HMDB03229 | **↑**^Δ^** | **↓**^Δ^** | /- | **↓**^Δ^** | /- |
| 10.98 | 328.2328 | C_22_H_32_O_2_ | - | Docosahexaenoic acid *^p,a^* | HMDB02183 | **↑**^Δ^** | /- | ^Δ^- | /- | /- |
| 11.01 | 729.5310 | C_40_H_76_NO_8_P | + | PC (14:0/18:2) *^p^* | HMDB07874 | **↓**^Δ^** | /- | /- | /- | /- |
| 11.14 | 304.2328 | C_20_H_32_O_2_ | - | Arachidonic acid.1 *^p,a^* | HMDB01043 | **↑**^Δ^** | /- | /- | **↓**^Δ^** | /- |
| 11.25 | 280.2329 | C_18_H_32_O_2_ | - | Linoleic acid *^p,a^* | HMDB00673 | **↑**^Δ^** | /- | /- | **↓**^Δ^** | /- |
| 11.92 | 282.2484 | C_18_H_34_O_2_ | - | Oleic acid *^p,a^* | HMDB00207 | **↑**^Δ^** | /- | /- | /- | /- |
| 12.22 | 304.8858 | C_20_H_32_O_2_ | - | Arachidonic acid *^u,a^* | HMDB01043 | **↑**^Δ^** | **↓**^Δ^** | **↓**^Δ^** | **↓**^Δ^** | /- |
| 12.42 | 809.6010 | C_46_H_84_NO_8_P | + | PC (22:4/16:0) *^p^* | HMDB08626 | **↓**^Δ^** | /- | /- | **↓**^Δ^** | /- |
| 12.44 | 757.5703 | C_42_H_80_NO_8_P | + | PC (14:1/20:1) *^p^* | HMDB07912 | **↓**^Δ^** | ^Δ^- | **↓**^Δ^** | /- | /- |
| 13.05 | 785.6016 | C_44_H_84_NO_8_P | + | PC (18:1/18:1) *^p^* | HMDB00593 | **↓**^Δ^** | /- | /- | /- | /- |
| 13.07 | 853.5694 | C_50_H_80_NO_8_P | + | PC (20:4/22:6) *^p^* | HMDB08452 | **↑**^Δ^** | /- | **↓**^Δ^** | **↓**^Δ^** | /- |
| 13.71 | 805.5695 | C_46_H_80_NO_8_P | + | PC (22:6/16:0) *^p^* | HMDB08725 | **↓**^Δ^** | **↑**^Δ^** | **↑**^Δ^** | **↑**^Δ^** | **↑**^Δ^** |
| 14.01 | 781.5694 | C_44_H_80_NO_8_P | + | PC (22:4/14:0) *^p^* | HMDB08623 | **↑**^Δ^** | **↓**^Δ^** | /- | **↓**^Δ^** | **↑**^Δ^** |
| 14.58 | 792.0765 | C_45_H_78_NO_8_P | + | PE (18:1/22:5) *^p^* | HMDB09077 | ↑^Δ^** | /- | /- | /- | ↑^Δ^** |
| 14.78 | 757.5698 | C_42_H_80_NO_8_P | + | PE (22:2/15:0) *^p^* | HMDB09549 | **↑**^Δ^** | **↓**^Δ^** | /- | **↓**^Δ^** | /- |

Notes: “Δ” indicates AUC area value ≥ 0.8, whereas “/” indicates AUC area value < 0.8; “*” and “**” indicates the Student’s *t*-test value of *p* < 0.05 and *p* < 0.01, and “–” indicates no statistically significant difference; Arrow “↑” indicates an increased level of metabolites; Arrow “↓” indicates a decreased level of metabolites; MI represents MI model group; H-CNS represents the H-CNS group; M-CNS represents the M-CNS group; L-CNS represents the L-CNS group. “a” indicates compounds confirmed with authentic standards. “p” indicates metabolite in plasma; “u” indicates metabolite in urine.
